# Evaluation of microalgae polysaccharides as biostimulants of tomato plant defense using metabolomics and biochemical approaches

**DOI:** 10.1038/s41598-020-78820-2

**Published:** 2021-01-13

**Authors:** Farid Rachidi, Redouane Benhima, Yassine Kasmi, Laila Sbabou, Hicham El Arroussi

**Affiliations:** 1 Green Biotechnology Center, MASCIR (Moroccan Foundation for Advanced Science, Innovation & Research), Rue Mohamed Al Jazouli Madinat Al Irfane, 10 100 Rabat, Morocco; 2 Microbiology and Molecular Biology Team, Center of Plant and Microbial Biotechnology, Biodiversity and Environment, Faculty of Sciences, Mohammed V University, 4 Avenue Ibn Battouta, B.P. 1014, Rabat, Morocco; 3 Agrobiosciences Program, University Mohamed 6 polytechnic (UM6P), Benguerir, Morocco

**Keywords:** Biochemistry, Biotechnology, Plant sciences

## Abstract

Microalgal polysaccharides (PSs) may be an effective elicitor agent that can efficiently protect plants against biotic stresses. In this study, wee investigates, the effect of PS obtained from microalgae and cyanobacteria (*D. salina MS002, P. tricorontum MS023, Porphyridium *sp. *MS081, Desmodesmus *sp.*, D. salina MS067 and A. platensis MS001*) on the biochemical and metabolomics markers linked to defense pathways in tomato plants. The phenylalanine ammonia lyase (PAL), chitinase, 1,3-beta-glucanase and peroxidase (POX) activities have been improved in tomato plants leaves treated by polysaccharides extracted from *P. triocnutum* (238.26%); *Desmodesmus* sp. (19.95%); *P. triocnutum* (137.50%) and *Porphyridium* sp. (47.28%) respectively. For proteins, polyphenols and H_2_O_2_, the maximum effect was induced by *D. salina 067* (55.01%), *Porphyridium *sp. (3.97%) and *A. platensis* (35.08%) respectively. On the other hand, Gas Chromatography-mass spectrometry (GC–MS) metabolomics analysis showed that PSs induced the modification of metabolite profile involved in the wax construction of tomato leaves, such as fatty acids, alkanes, alkenes and phytosterol. PS treatments improved the accumulation of fatty acids C16:3, C18:2 and C18:3 released from the membrane lipids as precursors of oxylipin biosynthesis which are signaling molecules of plant defense. In addition, PS treatment induced the accumulation of C18:0 and Azelaic acid which is a regulator of salicylic acid-dependent systemic acquired resistance. However, molecular and metabolic studies can determine more precisely the mode of action of microalgal polysaccharides as biostimulants/elicitors plant defense.

## Introduction

Plants in their environment are exposed to divers biotic and abiotic stresses^1^. Traditionally, synthetic pesticides and fertilizers are almost indispensable for the control of plant diseases and crop production. The excessive use of these synthetic chemical products is of global concern, because of their harmful effect on human health and the environment (European Commission 2012)^2,3^. In addition, the overuse of chemical products is the main cause of resistance development in pathogens^4^. In this context, the promotion of eco-friendly alternatives appeared necessary for the reduction of the use of chemicals in agriculture and hence of the attenuation of their environment effect^5,6^.

In the last decades, the sustainable agriculture has gained increasing interest on integrated crop management strategy where a promote the use of alternatives tools such as crop rotation, planting time, tillage and the use of biological products^3,7,8^. A lot of attention has been focused on the development of plant biostimulants to improve crop growth, yield and quality by enhancing plant nutrition, reducing abiotic stress impact and promoting plant growth^7,9,10^. Detailed reports are available on the induction of defense resistance in plants via a variety of biological inducers such as plant extracts, essential oils, bacteria and fungus, algal extracts and polysaccharides^11^. These elicitors have no direct toxic effect on pathogens and pests, but they have the ability to induce signaling pathways related to plant defense against biotic and abiotic stresses^1^, which makes them amenable as replacement for traditional agrochemicals in a sustainable crop production system. Algal polysaccharides are macromolecules with structural complexity linked to chemical groups with the high potential biological activities make algae polysaccharides an ideal bioresource of plant resistance inducers^12^.

Microalgae are photosynthetic eukaryotic and prokaryotic microorganisms with great biological and biochemical diversity, which have a potential as a promising bio-resource for different applications such as pharmaceutical, nutraceutical, food and feed, aquaculture, bioenergy and the environment^13–19^. In the agriculture field, the algal polysaccharide have a biostimulant activities of plant growth, nutrients uptake and biotic and abiotic stresses tolerance^16,20–24^. Recently studies, have demonstrated the biostimulant effect of microalgal extracts on the seed germination, seedling growth and yields of Lettuce and tomato plants^24–28^. However, until now, the use of microalgal extracts as biostimulants of natural plant defense mechanisms related to abiotic and biotic stresses is an undergoing initiation^24,28,29^. In the same way^24^, have demonstrated that *D. salina* exopolysaccharide have the potential to induce the metabolic and biochemical pathways related to natural plant defense in tomato plants under salt stress. Lipopolysaccharides and exopolysaccharides are essential structures maintained in pathogenic, non-pathogenic and saprophytic microorganisms, under the name of MAMPS/PAMPS (microbe-/pathogen-associated molecular patterns). These molecules are rapidly detected by the membrane receptors of the host plant, in order to induce various defense responses^30^.

Divers studies have demonstrated the potential of different extracts and metabolites—among them polysaccharides—of microalgae as biostimulants and biofertilizers of plant growth and crop production^12,26,29,31–34^. In the present study, we investigated polysaccharides Extracts of 6 Microalgae and Cyanobacteria species for their Bioelicitors effects on the different biochemical and metabolomics pathways related to the plants defense to biotic and abiotic stress of *Solanum lycopersicum L.* The aim of this research is to demonstrate the potential of microalgae polysaccharides as new bioactive materials for bioproduct development in agriculture.

## Results

### Microalgae culture and polysaccharides characterization

As shown in Table 1 microalgae growth differed between strains, *A. platensis* showed the fastest growth, followed by *D. salina MS067*, *D. salina MS002, Porphyridium *sp.*, P. tricornutum, and Desmodesmus *sp. with the biomass production of 1.22, 1.13, 0.91, 0.60, 0.45 and 0.42 g L^−1^ respectively (Table 1)*.* The choice of these strains for our project was based on the adaptation and the feasibility of their culture at large-scale. The microalgae polysaccharide content was recorded as 12,7%, 5.52%, 4.10% and 2.58% for *P. tricornutum, Porphyridium *sp.*, D. salina 002 and A. platensis* respectively, while their partial characterization indicated that they were composed of neutral sugars (5.0–35.350%), sulfates (4.051–11.541%), uronic acids (3.602–42.865%), proteins (2.701–22.068%), amino acids (0.017–0.206%) and β-glucans (0.008–0.698%). *P. tricornutum* exhibited the maximum production of polysaccharides (12.7%) composed of 32,3%, 6,75% and 0,698% of uronic acid, sulfate content and beta-glucan content respectively. Whereas *D. salina and Porphyridium *sp. polysaccharides exhibited the highest sulfate content with 11.5% et 10.4% respectively (Table1, Supplementary Fig. S1).

**Table 1:** Biomass production and composition of polysaccharides extracted from Moroccan microalgae.

Strains	Biomass (g L^−1^)	PS (%/biomass)	Total sugars (%/PS)	Sulfate (%/PS)	Uronic acid (%/PS)	Protein (%/PS)	Amino acid (%/PS)	Beta Glucan (%/PS)
*A. platensis*	1.22	2.590 ± 0.40	17.275 ± 1.05	5.306 ± 0.37	3.602 ± 1.58	2.746 ± 0.21	0.206 ± 0.0	0.149
*D. salina002*	1.13	4.103 ± 0.13	33.050 ± 0.92	11.541 ± 0.92	23.951 ± 2.62	4.202 ± 0.02	0.083 ± 0.0	0.008
*Porphyridium * sp.	0.6	5.529 ± 0.15	35.350 ± 0.05	10.427 ± 0.54	13.808 ± 5.45	2.701 ± 0.01	0.017 ± 0.0	0.108
*P. tricoruntum*	0.45	12.70 ± 2.66	5.003 ± 0.00	6.751 ± 0.59	32.331 ± 3.14	22.068 ± 0.13	0.019 ± 0.0	0.698
*Desmodesmus * sp.	0.42	7.2 ± 0.6	5.04 ± 0.00	5.27 ± 0.10	42.865 ± 10.4	11.850 ± 0.08	0.196 ± 0.0	0.043
*D. salina 067*	0.91	6.17 ± 0.44	5.0 ± 0.00	4.051 ± 0.24	23.951 ± 5.15	15.744 ± 0.09	0.181 ± 0.0	0.037

### Microalgal polysaccharides effect on tomato plants defense pathways

To investigate the effect of microalgae crude PSs, different enzymatic and biochemical markers were used in this study such as PRs proteins (Chitinase and 1,3-β,Glucanase) and PAL (Phenylalanine-Ammonia-Lyase) activities, ROS (H_2_O_2_) forms, peroxidase (POX) activity and polyphenols content.

### Microalgal polysaccharides effect on protein content, PAL, chitinase and 1,3-β,glucanase activities

**Figure 1:**
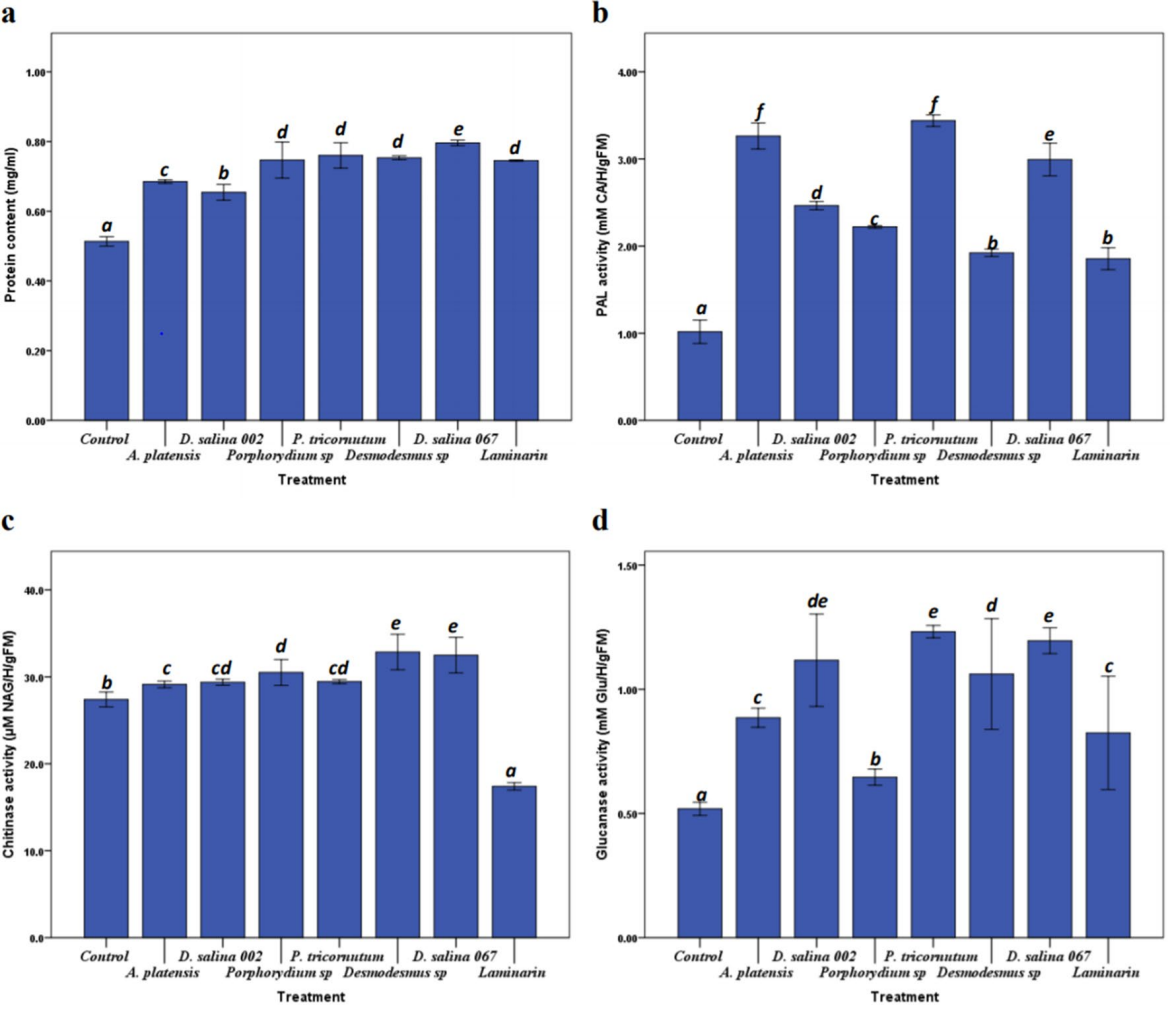
The effect of crude PS on protein content (**a**), PAL (**b**), chitinase (**c**) and β-1,3-Glucanase (**d**), activities. Tomato plants were injected with 1 mg mL^−1^ of PS. H_2_O and Laminarin (1 mg mL^−1^) were used as negative and positive controls respectively. Values are the mean (± SE) of five replicates. Different letters indicate significant difference between treatments (*p* < 0.05) according to Turkey-test, One-way ANOVA.

The results in Fig. 1a show that all PS treatments had a highly significant (*p* < 0.05) effect on protein content in treated plants compared to non-treated control plants. The maximum effect was induced by *D. salina 067* polysaccharides with 55.01% improvement compared to the control.

The enzymatic activity of PAL is highly significant (*p* < 0.05) in plants treated with different PS extracts (Fig. 1b). The percentages of minimal and maximal improvement (82.46% and 238.26%) in treated plants were induced by Laminarin and *P. triocnutum* polysaccharides respectively. In the same way, the chitinase activity showed the high significant (*p* < 0.05) enhancement in treated plants compared to control plant (Fig. 1c). The chitinase activity induced by polysaccharides from *Desmodesmus *sp., *D. Salina 067, Porphyridium *sp.*, P. tricornutum, D. salina 002 and A. platensis* was increased by 19.95%, 18.63%, 11.36%, 7.51%, 7.26% and 6.35% respectively compared to control plants (Fig. 1c). Results presented in Fig. 1d, shows significant (*p* < 0.05) effect of polysaccharides on 1,3-β,Glucanase activity compared to the control plants. It was also observed that the maximum percentage improvement (137.50%) was induced by *P. tricocnutum* polysaccharides *followed by D. salina MS067*and *D. salina MS002*, which had a highly significant (*p* < 0.05) effect compared to Laminarin-treated plants (positive control).

### Microalgal polysaccharides effect on peroxidase activity, H_2_O_2_ and Polyphenols

**Figure 2:**
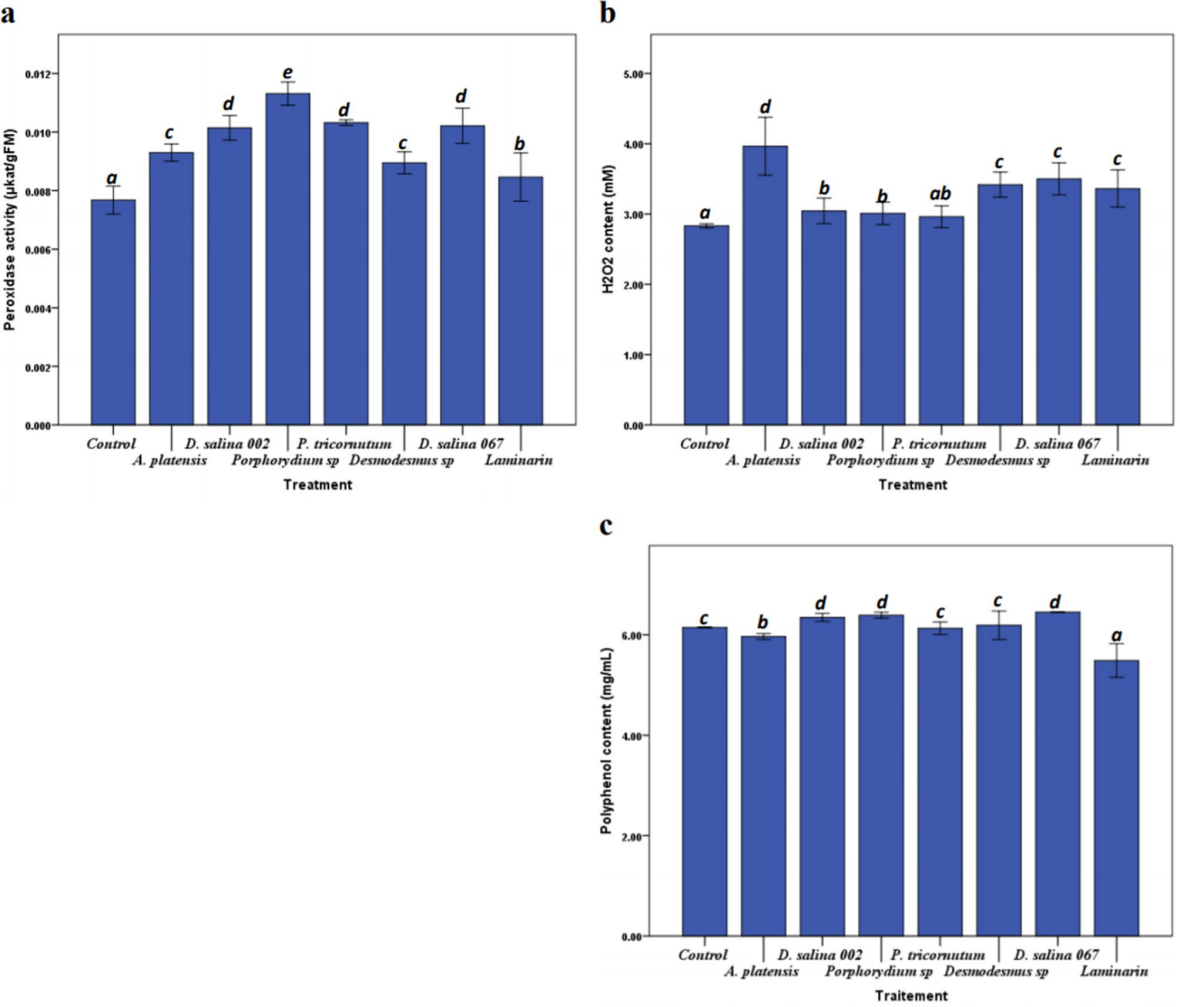
Peroxidase activity (**a**), H_2_O_2_ (**b**) and polyphenols content (**c**) in the tomato plants leaves after Polysaccharide treatment. Tomato plants were injected with 1 mg mL^−1^ of PS, and H_2_O or Laminarin (1 mg mL^−1^) as a negative and positive controls respectively. Values are the mean (± SE) of five replicates. Different letters indicate significant difference between treatments (*p* < 0.05) according to Turkey-test, One-way ANOVA.

The results presented in Fig. 2a, shows that the plants treated by PS of *Porphorydium *sp.*, P. triocnutum, D. salina 067* and *D. salina MS002* had a very significant (*p* < 0.05) effect on peroxidase activity compared to controls plants. The maximum improvement of peroxidase activity was noted in plants treated by *Porphyridium *sp. polysaccharides with percentage difference of 41% compared to non-treated plants. The evaluation of the effect of microalgae polysaccharides on oxidative burst was estimated by the determination of the amount of H_2_O_2_ accumulated in tomato plants leaves. Figure 2b, shows that all PS treatments except that of *P. triocuntum*, had a significant (*p* < 0.05) effect on H_2_O_2_ content in tomato plants leaves. On the other hand, *A. platensis* PS treatment had a much higher effect on H_2_O_2_ content compared to the Laminarin treatment -positive control- and with a percentage improvement of 35.08% compared to control plants. While, the polyphenol compounds in tomato plants leaves showed little improvement in plant treated by microalgae polysaccharides compared to control plants (Fig. 2c).

### GC–MS metabolomics analysis of tomato plants’ response to microalgae polysaccharide treatment:

**Figure 3:**
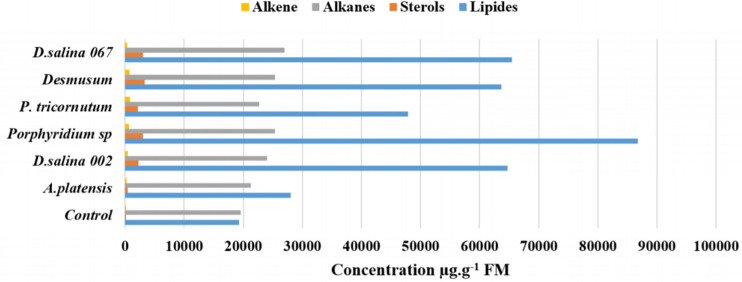
Metabolites profile of tomato plants leaves treated by polysaccharides. Tomato plants were injected with 1 mg mL^−1^ of PS, and H_2_O as Control.

For Metabolic fingerprinting, the GC–MS was used to identify metabolomic change in tomato plants under different microalgae polysaccharides treatment. The Fig. 3 shows a significant redistribution of several metabolites in treated plants such as lipids, sterols, alkanes, alkenes and other molecules. The lipids, sterols, alkanes and alkenes content in tomato leaves were improved by (31.22–77.79%), (79.40–97.11%), (8.20–27.75%) and (57.11–88.70) respectively compared to non-treated plants. It can be seen that the distribution of the metabolites was related to the nature of the polysaccharide. Also, the maximum biosynthesis of lipids, the sterols, alkanes and alkenes were induced by the PS extracts obtained from *Porphyridium *sp.*, Desmodesmus *sp.*, D. salina 067 and P. tricornutum* respectively (Fig. 3).

### Microalgae polysaccharides effect on lipid and sterol profiles in tomato plants:

Lipid profile analysis using GC–MS revealed 23 FAMEs dispersed over 3 categories of fatty acids namely: Very long chain fatty acids (VLCFA), unsaturated fatty acids (UFA) and saturated fatty acids (SFA). Fatty acids highly induced by the various PS treatments were linolenic acid (C18:3), palmitic acid (C16:0), roughanic acid (C16:3) and stearic acid (C18:0), with percentage of 50%, 30%, 5% and 10% respectively (Table 2). Meanwhile, VLCFAs included behenic acid (C22:0), tricosylic acid (C23:0), and lignoceric acid (C24:0) (Table 2).

**Table 2:** Lipids profile of tomato plants leaves treated by microalgae polysaccharides.

		*Control*	*A. platensis*	*D. salina 002*	*Porphyridium * sp.	*P. tricornutum*	*Desmodesmus * sp.	*D. salina 067*
**Lipids (µg g**^**−1**^**)**								
Azelaic acid	C9:0	41.92	146.66	197.80	119.48	147.91	200.17	167.70
Lauric acid	C12:0	97.49	395.19	563.56	478.36	322.19	409.64	431.97
Myristic acid	C14:0	73.63	326.23	453.98	439.97	355.99	457.66	402.26
Palmitic acid	C16:0	5843.54	6935.68	15,763.06	24,434.95	15,946.61	21,679.42	19,086.40
Roughanic acid	C16:3	1304.35	2578.48	4848.55	5445.68	2576.28	3408.04	4179.01
Margaric acid	C17:0	364.80	631.64	1183.71	2393.29	1046.90	1324.10	1375.93
heptadecatrienoate	C17:3	0.00	238.68	4121.15	0.00	0.00	0.00	0.00
Stearic acid	C18:0	1586.44	1506.31	3697.84	9229.94	3559.71	5338.57	5703.57
Linoleic acid	C18:2	107.59	0.00	0.00	0.00	290.99	0.00	0.00
Linolenic acid	C18:3	9389.95	14,068.73	31,142.12	40,959.72	21,391.22	27,192.53	30,833.82
Nonadecylic acid	C19:0	0.00	0.00	285.90	0.00	0.00	0.00	0.00
Arachidic acid	C20:0	164.04	330.37	603.88	934.11	606.33	980.57	810.08
Eicosatrienoic acid	C20:3	0.00	175.90	0.00	0.00	0.00	0.00	0.00
heneicosylic acid	C21:0	0.00	0.00	0.00	244.97	127.76	0.00	0.00
Behenic acid	C22:0	92.25	220.70	357.39	588.86	408.30	678.46	511.46
Tricosylic acid	C23:0	0.00	70.66	102.65	0.00	117.95	181.00	133.78
Lignoceric acid	C24:0	69.81	190.21	431.75	747.03	448.26	722.36	632.84
**Lipid derivatives (µg g**^**−1**^**)**								
9-Hexadecenoic acid, methyl ester		0.00	0.00	79.13	0.00	0.00	0.00	0.00
Nonanoic acid, 9-oxo-, methyl este		0.00	0.00	0.00	0.00	118.26	143.42	208.67
Pentanoic acid, 4-oxo-, methyl ester		142.98	213.71	0.00	0.00	0.00	961.30	0.00
Hexadecanoic acid, 2-hydroxy-, methyl ester		0.00	0.00	930.06	0.00	0.00	0.00	1010.16
Methyl 10-trans,12-*cis*-octadecadienoate		0.00	0.00	0.00	0.00	395.57	0.00	0.00
Methyl 9-*cis*,11-trans-octadecadienoate		0.00	0.00	0.00	794.14	0.00	0.00	0.00
Total		19,278.79	28,029.14	64,762.54	86,810.50	47,860.24	63,677.24	65,487.65
**Total (µg g**^**−1**^**)**								
VLCFA		162.06	481.56	891.79	1335.89	974.51	1581.82	1278.08
UFA		10,801.89	17,061.80	40,111.82	46,405.40	24,258.49	30,600.57	35,012.82
SFA		8171.86	10,272.07	22,749.74	38,275.07	22,113.41	30,390.14	27,977.91
**Sterols (µg g**^**−1**^**)**								
Stigmastan-3,5,22-trien		55.70	0.00	1032.85	0.00	0.00	0.00	0.00
Stigmasta-3,5-diene		39.04	74.84	875.03	1023.03	710.37	1017.92	997.53
Cholesta-3,5-diene		0.00	29.23	366.53	639.52	368.08	0.00	551.78
Stigmastan-6,22-dien, 3,5-dedihydro-		0.00	151.18	0.00	0.00	1129.49	0.00	1447.17
beta.-Sitosterol		0.00	204.77	0.00	0.00	0.00	0.00	0.00
Cholesta-6,22,24-triene, 4,4-dimethyl-		0.00	0.00	0.00	1353.16	0.00	0.00	0.00
Stigmasteryl tosylate		0.00	0.00	0.00	0.00	0.00	1747.20	0.00
Cholest-5-en-3-ol (3.beta.)-, nonanoate		0.00	0.00	0.00	0.00	0.00	516.56	0.00
Total		94.75	460.02	2274.40	3015.71	2207.95	3281.69	2996.47

Some changes in the fatty acid profile were also recorded after treatment with Microalgae PS (Table 2). PS treatment triggered the disappearance of 9-Hexadecenoic acid methyl esters which were present only in control plants, but induced the biosynthesis of new molecules such as Nonanoic acid, 9-oxo-, methyl ester, Pentanoic acid, 4-oxo-, methyl ester, Hexadecanoic acid, 2-hydroxy-, methyl ester, Methyl 10-trans, 12-*cis*-octadecadienoate and Methyl 9-*cis*, 11-trans-octadecadienoate.

On the other hand, microalgae PS treatment induced a remodulation of the distribution of sterol metabolites in tomato plant leaves. Generally, there was a disappearance of Stigmastan-3,5,22-trien and Stigmasta-3,5-diene in all treated plants compared to control plants. PS treatment induced the synthesis of new sterol metabolites namely: Cholesta-3,5-diene; Stigmastan-6,22-dien, 3,5-dedihydro-; beta.-Sitosterol; Cholesta-6,22,24-triene, 4,4-dimethyl-; Stigmasteryl tosylate and Cholest-5-en-3-ol (3.beta.)-,nonanoate. The synthesis of these latter depends on the composition of the PS extract used. For example, stigmasteryl tosylate, was highly accumulated (1747.20 μg g^−1^ FM) in plants treated with *Desmodesmus *sp. polysaccharides. Whereas, Stigmastan-6.22-dien, 3,5-dedihydro, Cholesta-6,22,24-triene, 4,4-dimethyl, Stigmastan-6, 22-dien, 3,5-dedihydro, Stigmastan-3,5,22-trien, and beta.-Sitosterol were induced in plants treated by *D. salina 067, Porphyridium *sp.*, P. tricornutum, D. salina 002 and A. platensis* polysaccharides respectively (Table 2).

### Microalgae polysaccharide effect on alkanes and alkenes profile

The results presented in Table 3 show that the effect of microalgae PS treatment on the profile of alkanes and alkenes in tomato plants. For Alkanes*,* heptadecane was accumulated to concentrations reaching 2866.68, 2673.91, 1743.36 and 1716.06 μg g^−1^ of fresh biomass of the treated plants with crude polysaccharides of *Desmodesmus *sp.*, Porphyridium *sp.*, D. salina MS067, D. salina MS002* respectively compared to control plants (351 μg g^−1^). While a new biosynthesis of eicosane had been induced in plants treated by PS from *D. salina MS067, P. tricornutum, D. salina MS002, A. platensis,* and *Desmodesmus *sp. with 2590.71, 1622.30, 1034.08, 840.30 and 161.03 μg g^−1^ of biomass plants respectively. Also, our results show synthesis of Very long chain alkane (VLCA), C25-C33 in plants treated with polysaccharides which were not observed in control plants. In the same way, PS treatment induced the accumulation of 7-Hexadecene and 1-Octadecene in the treated plants. The most synthesized alkene in treated plants was 1-Octadecene (503.18 μg g^−1^), induced by PS from *Desmodesmus *sp., Followed by 9-Nonadecene (399.36 μg g^−1^), then Z-8-Hexadecene (286 μg g^−1^) and cetene (279.08 μg g^−1^), these alkenes were induced respectively *by P. tricornutum, D. salina 067* and *Porphyridium *sp.

**Table 3:** The effect of microalgal polysaccharides on the biosynthesis and profile of alkanes and alkenes in tomato plants leaves.

		*Control*	*A. platensis*	*D. salina 002*	*Porphyridium * sp.	*P. tricornutum*	*Desmodesmus * sp.	*D. salina 067*
**Alkanes (µg g**^**−1**^**)**								
Heptadecane	C17	351.69	0.00	1716.06	2673.91	0.00	2866.68	1743.36
Octadecane	C18	31.56	0.00	234.35	0.00	1253.18	156.65	0.00
Eicosane	C20	0.00	840.30	1034.08	0.00	1622.30	161.03	2590.71
Heneicosane	C21	0.00	0.00	518.91	0.00	112.51	161.55	0.00
Docosane	C22	0.00	0.00	0.00	0.00	0.00	155.86	0.00
Tricosane	C23	0.00	0.00	0.00	0.00	0.00	0.00	177.99
Tetracosane	C24	35.77	709.66	0.00	1829.03	0.00	329.94	173.74
Pentacosane	C25	0.00	0.00	0.00	0.00	18.83	0.00	0.00
Hexacosane	C26	0.00	77.81	0.00	0.00	211.84	61.92	0.00
Heptacosane	C27	20.76	0.00	0.00	0.00	0.00	0.00	0.00
Octacosane	C28	0.00	179.19	449.78	161.42	0.00	155.86	188.26
Nonacosane	C29	0.00	217.76	0.00	74.07	0.00	0.00	0.00
Triacontane	C30	0.00	141.34	483.66	0.00	0.00	0.00	325.46
Hentriacontane	C31	0.00	0.00	0.00	988.70	0.00	2034.48	388.44
Dotriacontane	C32	0.00	177.29	0.00	295.25	463.09	513.11	416.66
Tritriacontane	C33	0.00	0.00	0.00	0.00	257.45	0.00	0.00
**Alkanes derivatives (µg g**^**−1**^**)**								
Trans-2,3-Epoxydecane		313.55	119.83	0.00	0.00	0.00	0.00	0.00
1-Chloroeicosane		0.00	62.39	0.00	0.00	0.00	0.00	0.00
Hexadecane, 1-iodo-		0.00	0.00	211.69	0.00	0.00	0.00	0.00
Octadecane, 1-iodo-		0.00	0.00	637.95	0.00	0.00	0.00	0.00
Bis-siloxane		28.36	0.00	0.00	0.00	0.00	0.00	0.00
Cycloeicosane		0.00	0.00	0.00	603.16	0.00	0.00	0.00
Cyclotriacontane		0.00	0.00	0.00	0.00	44.20	0.00	0.00
Cyclotetrasiloxane, octamethyl-		0.00	0.00	0.00	0.00	0.00	0.00	1587.31
Cyclopentasiloxane, decamethyl		0.00	0.00	0.00	0.00	0.00	0.00	412.08
Cyclohexasiloxane, dodecamethyl-		0.00	0.00	0.00	0.00	0.00	0.00	160.65
-17-((2R,5R)-5-Ethyl-6-methylheptan-2-yl)-		0.00	0.00	0.00	0.00	0.00	0.00	113.94
Total		781.69	21,263.07	24,023.98	25,363.04	22,720.90	25,334.59	27,016.10
**Alkenes (µg g**^**−1**^**)**								
7-Hexadecene		39.40	0.00	0.00	0.00	0.00	0.00	0.00
1-Octadecene		54.51	118.82	351.31	0.00	124.67	503.18	0.00
1-Pentadecene		0.00	100.15	72.24	0.00	0.00	0.00	58.63
1-Tetradecene		0.00	0.00	0.00	127.38	112.57	0.00	0.00
Cetene		0.00	0.00	0.00	279.08	194.58	234.31	0.00
2-Dodecen-1-yl		0.00	0.00	0.00	196.61	0.00	0.00	0.00
9-Nonadecene		0.00	0.00	0.00	0.00	399.36	0.00	0.00
Z-8-Hexadecene		0.00	0.00	0.00	0.00	0.00	0.00	286.04
Total		93.91	218.97	423.54	603.06	831.18	737.49	344.67

### Other metabolites

The results presented in Table 4, shows that the microalgae PS treatment induced the synthesis of new metabolites and the disappearance of other metabolites detected in untreated plants. All of the studied microalgae PS inhibited the biosynthesis of Isopropyl triacontyl ether, while they induced the synthesis of other molecules such as Neophytadiene, E-14-Hexadecenal, phenol, 4-methoxy-2-[2-(5-nitro-pyridinyl) diazenyl]- and Isopropyl octacosyl ether and others as shown in (Table 4).

**Table 4:** Effect of polysaccharides treatments on profile of others metabolites (µg g^−1^) in tomato plants.

Metabolites	*Control*	*A. platensis*	*D. salina 002*	*Porphyridium * sp.	*P. tricornutum*	*Desmodesmus * sp.	*D. salina 067*
Citric acid, trimethyl ester	0.00	0.00	0.00	0.00	0.00	0.00	293.84
2,4-Di-tert-butylphenol	25.54	0.00	0.00	0.00	0.00	0.00	128.33
E,E,Z-1,3,12-Nonadecatriene-5,14-diol	59.39	0.00	0.00	0.00	0.00	0.00	0.00
Isopropyl triacontyl ether	101.33	0.00	0.00	0.00	0.00	0.00	0.00
2(4H)-Benzofuranone, 5,6,7,7a-tetrahydro-4,4,7a-trim	0.00	70.54	0.00	0.00	73.48	80.41	104.24
Neophytadiene	0.00	406.57	0.00	0.00	0.00	0.00	0.00
alpha.-Tocospiro B	0.00	123.44	0.00	0.00	0.00	0.00	0.00
Tris(2,4-di-tert-butylphenyl) phosphate	0.00	38.64	0.00	0.00	162.00	100.49	75.05
E-14-Hexadecenal	0.00	0.00	295.24	0.00	0.00	0.00	0.00
2-Nitroso-1-naphthol-4-sulfonic acid	0.00	0.00	0.00	157.36	0.00	0.00	0.00
phenol, 4-methoxy-2-[2-(5-nitro-pyridinyl)diazenyl]-	0.00	0.00	0.00	1439.15	831.34	1358.66	0.00
3H-indole, 2-methyl-3-phenyl-	0.00	0.00	0.00	203.07	0.00	0.00	0.00
Phenol, 2,5-bis(1,1-dimethylethyl)	0.00	0.00	0.00	0.00	90.95	0.00	0.00
Diethyl Phthalate	0.00	0.00	0.00	0.00	91.48	0.00	0.00
1-(4-Fluorophenyl)-2-[(4-hydroxy-6 -methylpyrimidin-2-yl)thio]ethan-,	0.00	0.00	0.00	0.00	686.86	0.00	0.00
Methyl 2-octylcyclopropene-1-heptanoate	0.00	0.00	0.00	0.00	771.24	0.00	0.00
1,8-Dioxa-5-thiaoctane,8-(9-borabicyclo[3.3.1]non-9-yl)-3-(9-‘	0.00	0.00	0.00	0.00	138.55	0.00	0.00
4H-1,2,4-Triazole-3-thiol, 4-(2-phenylethyl)-5-(4-pyridyl)-	0.00	0.00	0.00	0.00	0.00	0.00	111.78
2-Propenoic acid, 3-(4-hydroxyphenyl)-, methyl ester	0.00	0.00	0.00	0.00	0.00	0.00	258.53
Carbonic acid, octadecyl 2,2,2-trichloroethyl ester	0.00	0.00	0.00	0.00	0.00	0.00	344.35
Isopropyl octacosyl ether	0.00	0.00	0.00	0.00	0.00	0.00	504.51
Cyclododecyne	0.00	0.00	0.00	0.00	0.00	359.98	0.00
Cyclooctene, 3-ethenyl-	0.00	0.00	0.00	0.00	0.00	448.70	0.00
Methyl 9-*cis*,11-trans-octadecadien	0.00	0.00	0.00	0.00	0.00	780.51	0.00
6-Nitro-2,3-dimethylpyrrolo[3,2-g]quinoline	0.00	0.00	0.00	0.00	0.00	93.51	0.00
Undec-10-ynoic acid, undec-2-en-1-yl ester	0.00	0.00	0.00	0.00	0.00	321.13	0.00
Protopine	0.00	0.00	0.00	0.00	0.00	1066.43	0.00
Dioncophyllin A	0.00	0.00	0.00	0.00	0.00	89.98	0.00
	0.00	0.00	0.00	0.00	0.00	0.00	0.00
Total	186.26	639.19	295.24	1799.58	2845.89	4699.80	1526.78

## Discussion

To further evaluate the effect of microalgae polysaccharides on plant tolerance against stress, a biochemical, enzymatic and metabolomics studies on treated plants compared to control plants were performed. In the present study, a bio-elicitor, laminarin, was used as a positive control for its potential to induce plant defense responses; improvement of resistance to viral, fungal and bacterial infections, induction of accumulation of salicylic acid (SA) and induces expressions of ethylene (ET)- and SA-dependent PR proteins^35,36^. In general, it was observed in this study that certain polysaccharides treatments had a similar effect compared to laminarin, however, we see that other treatments have a highly significant effect on studied parameters. For instance, all the treatments exhibited great effect on chitinase, peroxidase, and polyphenols; whereas all treatments except *D. salina* 067 had great effect on PAL activity. On the other hand, polysaccharides extracted from *P. tricornutum* and *D. salina* induced significantly Glucanase activity; and *A. platensis* polysaccharides enhance H2O2 production. From these results, it can be concluded that microalgae PS can be good candidates for the induction of plant defense. The activities of PAL, POX, 1,3-β Glucanase and chitinase activities and H_2_O_2_ content are frequently studied in plant defense research and are known to correlate with the occurrence of induced resistance, as well as defending the host plant against microbial phytopathogens^37^.

**Figure 4:**
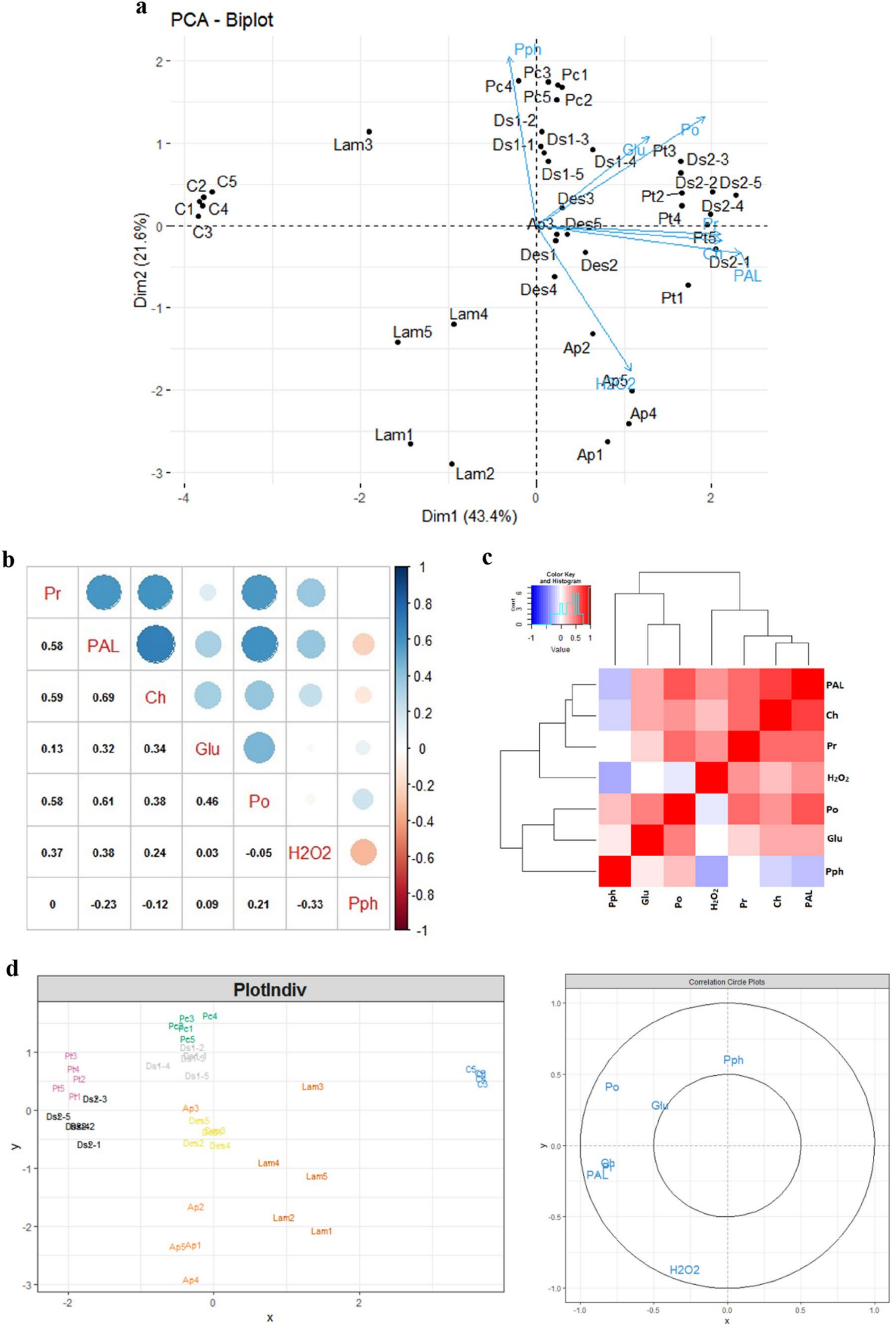
Biplot of principal component analysis of variables and treatments (**a**); Correlation between variables (**b**); Heatmap hierarchal clustering of studied variables (**c**); PLS-DA for biochemical variables and treatments (**d**). C: Control; A.p: A.platensis; P*.c: Porphyridium *sp.*; P.t: P. tricornutum; D.s1: D. salina 002; D.s2: D. salina 067; Des: Desmodesmus *sp.*; Lam: Laminarin; Pr: protein; PAL; Phenyl-alanine amonia-lyase; Ch: Chitinase; Glu: Glucanase; PO: Peroxidase; Pph: Polyphenol.*

Our result showed a strong positive correlation between peroxidase (POX) and PAL activities in response to microalgae polysaccharides treatment (Fig. 4a,b). These enzymes are key defense proteins related to plants resistance to diseases^38^. PAL, is the key enzyme of the phenylpropanoid synthesis pathway, and increase in PAL activity is accompanied by the biosynthesis of active metabolites such as Phytoalexins, lingnins, flavonoids, phenolics compounds and salicylic acid^39–41^. Another study has shown seaweeds algino-oligosaccharides could induce the enzyme activities of PAL and POX in rice plants as defense against *Magnaporthe grisea*^42^. Similarly, the treatment of Olive and Tomato plants by Ulvan significantly induced PAL activities against Verticillium wilt and *lycopersicicum* respectively^43,44^. Other studies concluded that there is a correlation and synergy between the stimulation of PAL and POX activities and the induction of tomato resistance to *Fusarium oxysporum f*, the causative agent of wilt disease, after plant treatment by *Pseudomonas fluorescens* Pf1 isolates^45^.

In response to a pathogen attacks, plants accumulate reactive oxygen species (ROS), the main chemical element of oxidative bursts^46^. Faced with the oxidative burst induced by a high accumulation of H_2_O_2_, plants have mechanisms that allow them to keep the H_2_O_2_ levels in homeostasis. The latter is controlled by detoxifiers or scavenging enzymes including; SOD (Superoxide dismutase), CAT (Catalase), APX (Ascorbate peroxidase) and POX (peroxidase)^47–49^. These enzymes coordinate the elimination of different ROS^50^. This explains our result, which revealed a negative correlation between peroxidase activity and H_2_O_2_ accumulation (Fig. 4a,b). That is to say that in the first step, the PS treatment induced the synthesis of H_2_O_2_, then at a certain time, H_2_O_2_ levels reached a high concentration which induced the synthesis of POX, in our case, Guaiacol peroxidase, which is found in different cell compartment, capable of producing phenoxy compounds by the reduction of H_2_O_2._ The polymerization of phenoxy compounds lead to the formation of cell wall components such as lignin^51^. On the other hand, peroxidases are involved in other physiological processes, namely the synthesis of phytoalexins, the formation of suberin, cross-linking of cell wall components and the synthesis of nitrogen species reagents (RNS) and the induction of the hypersensitive response, which is the programmed death of the host cells in the site of infection, for the limitation of pathogen propagation and the induction of the pathogenesis related proteins^52^. The transformation of phenolic compounds by peroxidases also explains our results which we noted that the high accumulation of POXs -after microalgae polysaccharides treatment- accompanied by a low accumulation of phenolic compounds in treated plants (Fig. 2a,c).

glucanase (Glu) and chitinase (Chi) are PR proteins which have the first enzymatic attacks of the plant against pathogens by degrading β-1,3-glucan and chitin present in the fungal cell wall, thus stimulating host defense responses^38^. These two enzymes intervene in the cases of the wounds and the infections by phytopathogens separately or in combination by reinforcing the defense of the plant against the stresses^53^. Our results showed that there is a strong accumulation of PR Proteins; Chitinase and Glucanase with a highly significant positive correlation (Fig. 4a,b). This correlation explains why the PS treatments are able to induce both the acquired systemic resistance (SAR) and the induced systemic resistance (ISR). The accumulation of chitinase is accompanied by the overexpression of its gene (PR3) which occurs in the jasmonic acid and ethylene compounds linked to the ISR. The induction of glucanase is ensured by the overexpression of its gene (PR2). The latter, the SA-dependent marker gene, is involved in the induction of SAR^54–56^. Several studies have shown that there is a great synergy between the accumulation of these two hydrolytic enzymes and the induction of plant defense by pathogens. The study done by^57^, showed that treatment of tobacco plants with sulfated Fucan Oligosaccharides which are reported as elicitors of the local and systemic response against *Tobacoo Mosic Viruss,* strongly induced the synthesis of Glucanase and Chitinase. In addition, the enhancement of Chitinase and Glucanase activities after treatment of tomato plants with *Pseudomonas fluorescens Pf1* induced their resistance to *Fusarium oxysporum f. *sp.* Lycopersici*^45^.

The PCA analysis revealed that the first three components accounted for 43.4%, 21.6%, and 12.1% of the variance, for a total of 77% (Fig. 4a). We believe that the first two major components provided sufficient information to corroborate the key findings presented here. Generally, the correlation is positive between the different variables (Protein, PAL, Glucanase, Chitinase and Peroxidase) (Fig. 4a,b). Hierarchical clustering spawned two large groups; Group 1: which contains proteins, Chitinase PAL and H_2_O_2_; and Group 2: which contains Glucanase, Peroxidase and Polyphenols (Fig. 4c). PLS-DA analysis generated some specificity between the type of treatment used and the type of variable induced. In this sense, it was found that the protein level and the glucanase and PAL activities were induced more by polysaccharides extracted from *P. tricornutum* and *D. salina 067*. In addition, the enzymatic activities of peroxidase and chitinase and the Polyphenols content were stimulated more by the polysaccharide-based treatment of *Porphyridium *sp. and *D. salina 002*. However, it can be seen that the accumulating effect of H_2_O_2_ is induced by *A. platensis.* Polysaccharides extracted from *Desmodesmus *sp. have a similar effect on all studied variables (Fig. 4d). This moderation of induction of enzymatic activities by microalgal PSs has prompted us to hold the combination of treatments as a perspective for the study of their effect on plant defense pathways.

The analyses, ACP-Biplot, correlation and Heatmap clustering of the metabolic variables, reveal (1): the correlation of the variables studied; (2): the specificity between the metabolite induced vis-à-vis the treatment used; (3): treatments inducing similar biochemical parameters as well as metabolomics parameters. The variables chosen for this analysis were: C9:0 (Azelaic acid), C12:0 (Lauric acid), C14:0 (Muristic acid), C16:0 (Palmitic acid), C16:3 (Roughanic acid), C17:0 (Margaric acid), C18:0 (Stearic acid), C18:3 (Linolenic acid), C20:0 (Arachidic acid), C22:0 (Behenic acid), C23:0 (Tricosylic acid) C24:0 (Lignoceric acid), VLCFA, UFA, SFA, Total Lipid, Total Sterol, Total Alkane, and Total Alkene.

**Figure 5:**
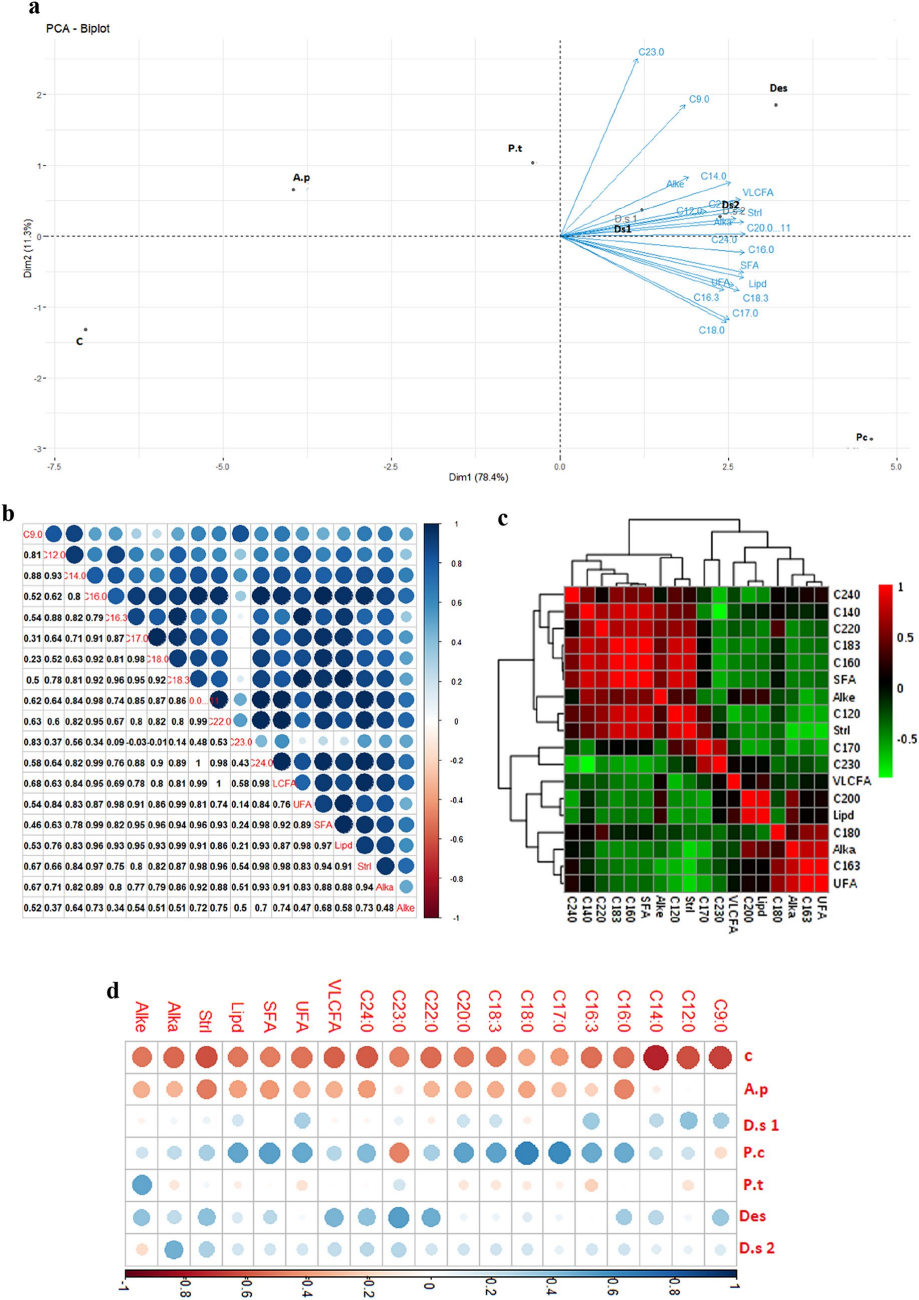
PCA-Biplot of metabolites and treatments (**a**), Correlation between metabolites (**b**), Heatmap Heatmap hierarchal clustering of metabolomics variable (**c**), and Heatmap of metabolites and treatments (**d**), after plant treatment by microalgae PS extract. *C: Control; A.p: A. platensis; P.c: Porphyridium *sp.*; P.t: P. tricornutum; D.s1: D. salina 002; D.s2: D.salina 067; Des: Desmodesmus *sp.

The results in (Fig. 5) show that in general the metabolomics variables are positively correlated with each other (Fig. 5a,b). Heatmap, in Fig. 5d, shows that the polysaccharide treatments from *Porphorydium *sp.*, Desmodesmus *sp.*, D. salina* 067 have a significant effect on the induction of the synthesis of different metabolites, followed by *D. salina* 002. On the basis of biochemical and metabolomics results, it can be seen that treatments based on *Porphorydium *sp.*, P. tricornutum, Desmodesmus *sp.* and D. salina 067* have a great potential to induce the different parameters studied.

The cuticle is composed of cutin polyester and cuticular wax; it allows plants’ resistance to biotic and abiotic stresses^58^. Generally, the cutin polymer is formed primarily of esterified and oxygenated fatty acids (FA) linked to C16 and C18^59^. Cuticular wax, which is composed of mixed complexes, contains mainly a variety of alkanes, primary and secondary alcohols, aldehydes, ketones and esters which are derived from very long chain fatty acids (C20–C34)^60^. All this explains our study findings, a strong accumulation of two precursors (fatty acids, C16 and C18) of VLCFA (C20–C24) and VLCA (C24–C33), induced by microalgae polysaccharide treatments. The strain that induced the maximum synthesis of two precursor’s palmitic acid C16:0 (9.781 μg mL^−1^) and stearic acid C18:0 (3.694 μg mL^−1^), was *P. creuntum*. Meanwhile, the appearance of VLCA (C24–C33), explains that VLCFAs (C25–C34), were transformed totally during the synthesis of VLCA (C24–C33).

Our results show that there is a positive correlation between the synthesis of (C16, C18), VLCFAs and VLCAs. This correlation is explained by the transformation of C16 and C18—through the elongation pathway—into VLCFAs^60^. The latter undergo an acyl-reduction and a decarocarbonylation—via the VLCAs—for the transformants into wax esters and ketones respectively. Then the latter are transported to the cuticle^60^. Among the main roles of the cuticle; it is a component that protects plants against desiccation and blocks the penetration of toxins and even pathogens^61,62^. The work done by^63^, showed that *Arabidopsis* plants exposed to water stress induced high synthesis content of cuticular wax accompanied with a very high accumulation of VLCAs, suggesting that the alkane biosynthesis plays an important role in abiotic stress tolerance. The cuticular permeability increases when the biosynthesis of VLCAs decreases^64^. Our results are comparable to that of^29^, who reported that *C. reinarditi and C. sorokiniana* polysaccharides significantly induced the synthesis of C16, C18, VLCFAs and VLCAs in tomato plants.

Plants, when infected by pathogens, trigger a series of metabolic redistributions that potentially contribute to induced tolerance or even increase sensitivity^65^.

Polyunsaturated fatty acids (PUFAs) are the primordial components of the plasma membrane. Their release is achieved by lipases after pathogen attack^66^. These FAs play a major role in plant-pathogen interactions; acting directly as free fatty acids or as fatty acid-derivatives (oxilipins)^66^.

In our study, increased synthesis of C18:2 as PUFAs in tomato plants after treatment with *Desmodesmus *sp. PSs, is similar to the results of^67^ which showed that increase in C18:2 synthesis in avocado fruits elicits increased resistance to the fungal pathogen, *Colletotrichum gloesporioides*. In our results we also see a strong accumulation of PUFAs; Roughanic acid (C16:3) and Linoleic acid (C18:3) after treatment with different PSs treatments. The maximum values of these two FAs are (2.180 μg mL) and (16.395 μg mL^−1^) respectively, obtained at treatment with *Porphyridium *sp. Trienoic fatty acids, are PUFAs that play a crucial role in adapting plants to abiotic and biotic stress. A study by^68^ showed that the high accumulation of linoleic acid (C18:3) in *Arabidopsis* leaves after inoculation with *Pseudomonas syringaepv. tomato* DC3000 (avrRpm1), activates NADPH-Oxidase, which is responsible for the synthesis of reactive oxygen species ROS^68^.

The strong accumulation of C18:3 in plants treated with PSs was accompanied by the accumulation of azelaic acid (AA, C9:0). Also, there was an induction of C18:2 synthesis in treated plants compared with control plants. Our results are similar to that of^29,69^, who demonstrated that after 24 h of treatment with microalgae polysaccharides, leaf content of C18:1, C18:2 and C18:3 was increased, leading to an induced accumulation of azelaic acid. These three unsaturated octadecanoic are azelaic acid (AA) precursors. Contact of host plants with a pathogen induces their defragmentation from the plasma membrane by phospholipases. After this, AGs undergo oxidation at carbon-9 to synthesize AA (71–74). The latter has been shown to induce local and systemic acquired resistance (SAR) in Arabidopsis plants against *Pseudomonas syringae* via the induction of salicylic acid accumulation^69,70^.

Phytosterols play a key role in plant innate immunity against bacterial pathogens by regulating nutrient efflux into the apoplast^71^. Stigmasterol are phytosterols derived from β-sitosterol by the cytochrome P450 CYP710A1 via C22 desaturation^65^. In our results, we observed that the sterol metabolites induced by the different PS extracts are Stigmasta-3,5-diene, Cholesta-3,5-diene and Stigmastan-6,22-dien, 3,5. The accumulation of stigmasterol was higher in plants treated with PS extracts compared to control plants. These results have been confirmed by the Wang’s study^71^, who reported that phytosterols play a vital role in plant defense against pathogenic bacteria through nutrient flow regulations from the cytoplasm to the apoplastic space. During the invasion of plants by pathogenic bacteria, the virulence factors of the bacterium block the expression of AtCYP710A1 gene (gene responsible for transformation of β-Sitosterol into Stigmasterol), thus repression of the production of stigmasterol., all its going to induce a reduction of flow of nutrients from cytoplasm to the host cell's appoplast^71^.

## Conclusion

In general, microalgae polysaccharides have a bio-elicitor effect on certain biochemical and metabolic pathways of tomato plant defense. In this study PS treatment induced the accumulation of ROS (H_2_O_2_) and the antioxidant activity of (POX). PS treatments also improved PAL activity, a key enzyme in the activation of the phytoalexin pathway. Pathogenesis-related proteins, such as β-1.3-Glucanase and Chitinase, which are key protein markers for plant defense against biotic stresses were also induced. Moreover, metabolomics study revealed that PS treatment induced the modification of metabolite profiles in tomato plant leaves. Induced metabolites included C18:2 and C18:3 and Azelaic acid which is a regulator of salicylic acid-dependent systemic acquired resistance. On the other hand, treated plants by PS exhibited higher accumulation of C16:0 and C18:0, VLCFAs and VLCAs which are metabolites linked to the strengthening of the cuticle by wax synthesis in order to inhibit the penetration of toxins from pathogens. These metabolites are also involved in the synthesis of sphingolipid complexes, the basal unit of the membrane lipid bilayer. Finally, PSs also induced the accumulation of sitosterol and stigmasterol derivatives. Sitosterol and stigmasterol play a key role in regulating nutrient flow to the apoplast, making plants resistant to pathogens. Meanwhile, Principal Component Analysis (PCA) showed that PSs from *P. tricoruntum MS023* and *D. salina MS067* significantly improved chitinase and PAL activities and proteins content. Whereas, the PS treatments of *Porphyridium *sp.* MS081* and *D. salina MS002* enhanced the POX and β-1,3-glucanase activities and Polyphenols content. On the basis of biochemical and metabolomics results, it can be seen that treatment with polysaccharides obtained from *Porphorydium *sp.*, P. tricornutum, Desmodesmus *sp.* and D. salina 067* have a great potential to induce the different studied parameters. This moderation of induction of enzymatic activities by microalgal PSs has prompted us to hold the combination of treatments as a perspective for the study of their effect on plant defense pathways.

Our results show that polysaccharide treatments based on microalgae can be used as a biotechnological alternative in sustainable agriculture to protect plants against pathogens and abiotic stresses and to also minimize the excessive use of chemical pesticides.

## Materials and methods

### Microalgae culture conditions and growth measurements

Microalgae strains used in this study (freshwater: *A. platensis MS001, seawater: D. salina MS002, P. tricornutum MS023, Porphyridium *sp.* MS081, Desmodesmus *sp.* and D. salina MS067*) were maintained in the microalgae collection at MAScIR (Moroccan Foundation for Advanced Science, Innovation and Research). These strains were cultivated in Walne’s medium with pH 8.2 at 25 °C for seawater strains *and A. platensis* in Zarrouk medium at pH 9 and 30 °C. All cultures were grown in 500 mL Erlenmeyer’s in orbital shaker at 130 rpm with continuous illumination (150 ± 10 μmol m^−2^ s^−1^). The biomass was harvested after 24 days of culture by centrifugation at 4200 rpm for 10 min and rinsed twice with a ammonium formate solution of 30 g L^−1^. The biomass is stored at -20 °C.

### Polysaccharides production and partial characterization

1 g of dry microalgae biomass powder was suspended in 40 mL of distilled water and incubated at 90 °C for 2 h with stirring. The mixture was centrifuged at 4000 rpm for 15 min. After recovering the supernatant, the extraction process was repeated twice, and then the supernatants were mixed with two volumes of absolute ethanol, stirred vigorously and left overnight at 4 °C. The precipitated polysaccharides were recovered in the pellet by centrifugation at 11,000 rpm for 10 min. The PS pellet was washed three times with absolute ethanol and lyophilized^72^. The total neutral sugar and uronic acids contents of the crude PS were determined according to Blumenkrantz and DuBois methods respectively^73,74^. Sulfate content in crude PS was determined by the barium chloride-gelatin method^75^. The protein content was determined according to the Bradford method using crystalline bovine serum albumin (BSA) as standard^76^.

### Microalgae polysaccharides effect on tomato plant defense

#### Bioassays for tomato growth and treatments

In this set of experiments, we used *Solanum lycopersicum L. Var. Jana F1* (tomato). Seeds (purchased from “Syngenta Morocco”) were surface-disinfected using sodium hypochlorite solution (1%) containing 10 µL of Tween-20 for 20 min and then rinsed with sterile water.

Sterile seeds were germinated on Murashige and Skoog (MS) + agar medium in magenta boxes, in the dark for 4 days and in the light for 3 days and the seedlings were transplanted into 12 cm diameter pots containing a mixture of sandy soil/peat (60/40, w/w). The tomato seedlings were grown in a phytotron at 26 °C, 16 h/8 h Light/dark cycle and 240 μmol m^−2^ s^−1^ illumination intensity.

Plants were irrigated every day with 10 mL of water, alternated twice a week with a nutrient solution containing: 8 μM MnCl_2_, 0.5 μM CuSO_4_·5H_2_O, 1.4 μM ZnSO_4_, 46 μM H_3_BO_3_, 0.25 μM Na_2_MoO_4_·2H_2_O, 0.6 μM Fe-EDTA, 4.1 mM KNO_3_, 0.9 mM K_2_SO_4_, 1 mM MgSO_4_·7H_2_O and 1.5 mM KH_2_PO_4_^77^.

The polysaccharide treatments were prepared with a ratio of 0.1% PS in distilled water. The pH was adjusted to 6 using HCl or NaOH diluted to 1 M.

The injection was performed using a microsyringe above the cotyledon leaves with a volume of 10μL of the 0.1% PS solution. 30 days after transplantation, the plants were injected with 10 μL of 1 mg mL of microalgae polysaccharides, 10 μL of Laminarin diluted to 1 mg mL^−1^ as the positive control) and 10 μL of distilled water (dH_2_O) for the negative control. 24 h after treatment, leaves were harvested according to treatment groups and grinded in liquid nitrogen. The samples were then stored at − 80 °C.

#### Biochemical and enzymatic parameters

##### Extraction and enzyme activity assay

100 mg of the tomato plant leaf biomass was homogenized in 1 mL of 100 mM Tris–HCl (pH 8.5) containing 14 mM of β-mercaptoethanol. The mixture was centrifuged at 13,000 rpm and 4 °C for 30 min. The supernatant, which contains the proteins was collected. Protein assay is determined according to Bradford method by using Bovine Serum Albumin (BSA) for the standard curve^76^.

The activity of phenylalanine ammonia-lyase was determined according to the modified methods previously described by Mozzetti and EL Modafar^78,79^.

The chitinase activity was determined according to modified Pegg method^80^.

Glucanase activity based on Chandrasekaran method^81^. The ground biomass was homogenized in 2 mL of 0.1 M Tris–HCl buffer (pH 7) containing 1% v/v of polyvinylpolypyrrolidone. The homogenate was centrifuged at 12,000 rpm for 15 min at 4 °C. The supernatant was recovered in 1.5 mL Eppendorf tubes and stored at − 20 °C. The reaction mixture contained 50 μL of the enzymatic extract, 50 μL of laminarine (10 mg mL^−1^) prepared in 50 mM of sodium acetate buffer, (pH 5.0) and incubated at 37 °C for 1 h on a heat block. The reaction was stopped by the addition of 1.5 mL of dinitrosalicylic acid (DNSA) and the mixture was incubated at 100 °C for 5 min a heat block. Absorbance was measured at 530 nm and 1,3-glucanase activity was calculated using the standard glucose curve. The activity of Glucanase was expressed as µM *N*-Acytelglucosamine H^−1^ g^−1^ FM.

The POX activity was carried out, following the method described by Chakraborty^82^. The substrate was prepared with 5 mL of 1% guaiacol, 5 mL of 0.3% H_2_O_2_ mixed in 50 mL of 0.05 M sodium phosphate buffer (pH 6.5). The reaction mixture was prepared with 2.95 mL of the substrate and 0.05 mL of the enzyme extract and the absorption change was measured at 470 nm for 3 min. POX activity was determined by the increase in the absorbance due to guaiacol oxidation and expressed as change in the absorption of the reaction mixture min^−1^ mg^−1^ of protein (E = 26.6 mM^−1^ cm^−1^) µkat g^−1^ FM.

The H_2_O_2_ determination according to Sanders method^83^. Tomato plants were harvested, immediately grinded in liquid nitrogen and stored at − 80 °C until H_2_O_2_ determination assay. The frozen grinded leaf biomass (150 mg) was directly homogenized in 1 mL solution containing 0.25 ml Trichloroacetic acid (TCA) (0.1% (w:v)), 0.5 mL KI (1 M) and 0.25 ml 10 mM potassium phosphate buffer (10 mM, pH adapted to studied tissue) at 4 °C for 10 min. At the same time, for every sample, a control was prepared with H_2_O instead of KI for tissue coloration background. Good care was taken to protect samples and solutions from light. The homogenate was centrifuged at 12,000 rpm for 15 min at 4 °C. H_2_O_2_ content was measured at 350 nm sing a UV/VIS spectrophotometer. A calibration curve obtained with H_2_O_2_ standard solutions prepared in 0.1% TCA was used for quantification.

The total phenolic content in tomato leaf extracts was determined by using Folin-Ciocalteu reagent^84^.

#### Metabolomics analysis

To investigate the effect of microalgae polysaccharides on the redistribution of metabolites in tomato plants, GC–MS metabolomics analysis in this study according to Kamthan method^85^ with some modifications. The metabolites analyzed are lipids, alkanes, alkenes, sterols and other metabolites.

300 mg of frozen leaf biomass was added to glass vials with cap. 10 µL of internal standard, Dodecane (0.75 g mL^−1^) was added to each tube, to which 4 mL of chloroform and methanol mixture with a ratio of (v/v; 2/1) was also added. The mixture was vortexed and incubated at 85 °C for 2 h (2 h on heat block), vortexed and then incubated at 60 °C in an ultrasound bath for). After this, 1 mL of H_2_O was added to the vials and the mixtures were thoroughly vortexed. The organic phase after phase shift was recovered by using a separating funnel and dried by evaporation with nitrogen gas. For acid transesterification, 500 µL of Methanol/sulfuric acid (6%, v/v) was added to the dried organic phase residue. The mixture was incubated on a heat block for 2 h at 85 °C, and then for 1 h in an ultrasound bath. After that, the nitrogen gas was used to drying the mixture, then 250 µL and 750 µL of H_2_O and Chloroform respectively was added to the mixture. The lower phase was then collected using a separating funnel.

For the metabolomic analysis of volatiles compounds, the gas chromatography (GC) (Agilent 7890A Series) coupled to mass spectrometry (MS) was used in this study. 4 μL of the samples were injected into the 123-BD11 column (15 m × 320 μm × 0.1 μm) by 1/4 split mode using helium as carrier gas at 3 mLmin^−1^. The temperature was 230 and 150 °C in ion source and quadrupole respectively, and oven temperature program was started at 30 °C and finished at 360 °C. The NIST 2014 MS Library was used for molecules identification.

### Statistical analysis

The R software (R Core Team 2013)^86^ was used to statistical analysis, graphical generation and corrplot and ggplot (Hadley Wickham 2016)^87^ packages integration. To determine the overall biochemical and metabolomics parameters variation, we used The Principal Component Analysis (PCA) and partial least squares-discriminant analysis (PLS-DA) were used studied the variation of metabolomic and biochemical parameters. The correlation between treatment and variables were studied by prcomp function and MixOmics package. The statistical analysis was studied using SPSS, one-way ANOVAs by Tukey HSD test.

## Supplementary Information

Supplementary Figure S1.
