# Supplementary material for: Evaluation of microalgae polysaccharides as biostimulants of tomato plant defense using metabolomics and biochemical approaches
Source: Sci Rep. 2021 Jan 13;11:930. doi: 10.1038/s41598-020-78820-2 (PMC7806925; doi:10.1038/s41598-020-78820-2)
Supplement: Supplementary file 1 — Supplementary Figure S1. [file 41598_2020_78820_MOESM1_ESM.docx]

**Evaluation of microalgae polysaccharides as biostimulants of tomato plant defense using Metabolomics and Biochemical approaches.**

**Farid Rachidi^1, 2^, Redouane Benhima^1^, Yassine Kasmi^1^, Laila Sbabou^2^, Hicham El Arroussi^1,3*^**

^1^ Green Biotechnology Center, MASCIR (Moroccan Foundation for Advanced Science, Innovation & Research)- Rue Mohamed Al Jazouli – Madinat Al Irfane, 10 100 Rabat – Morocco).

^2^ Microbiology and Molecular Biology Team, Center of Plant and Microbial Biotechnology, Biodiversity and Environment, Faculty of Sciences, Mohammed V University, Rabat,4 Avenue Ibn Battouta B.P. 1014 RP, Rabat. Morocco

^3^Agrobiosciences program, University Mohamed 6 polytechnic (UM6P) Benguerir, Morocco

*Corresponding author: [h.elarroussi@mascir.m](mailto:h.elarroussi@mascir.m)a, +212676833559

**Supplementary Information**

Supplementary Figure S1

|  |
| --- |
| **Figure S1.** Growth profile of algal cultures. The microalgae were cultivated in 500 mL Erlenmeyer containing Walne’s medium with pH 8.2 at 25°C for seawater strains*. A. platensis* was cultivated in Zarrouk medium at pH 9 and 30°C. All cultures were grown in their respective culture medias under agitation on an orbital shaker at 130rpm with continuous illumination (150 ± 10 μmol m^−2^ s^−1^) for 24 days. |
